# Transient asymptomatic pulmonary opacities in a patient with MET exon 14 skipping non‐small cell lung cancer: A case report

**DOI:** 10.1111/1759-7714.14831

**Published:** 2023-02-22

**Authors:** Satoshi Ikeo, Naoaki Yasuda, Yuki Sakai, Yasuyuki Hayashi, Akihiko Sokai, Toshiyuki Iwata, Takashi Nishimura

**Affiliations:** ^1^ Department of Respiratory Medicine Kyoto Katsura Hospital Kyoto Japan; ^2^ Department of Respiratory Medicine, Graduate School of Medicine Kyoto University Kyoto Japan

**Keywords:** lung cancer, MET exon 14 skipping, tepotinib, transient asymptomatic pulmonary opacities

## Abstract

Mesenchymal epithelial transition factor receptor (MET) tyrosine kinase inhibitors (MET‐TKIs) have been approved for the treatment of non‐small cell lung cancers with MET exon 14 skipping mutations. Transient asymptomatic pulmonary opacities (TAPOs) associated with epidermal growth factor receptor (EGFR)‐TKIs have been reported. Here, we report a case wherein ground‐glass opacities (GGOs) appeared during the course of treatment with tepotinib, a MET‐TKI, but spontaneously resolved with drug withdrawal, after which treatment was resumed with a reduced dose. Although there have been no reports of TAPOs with MET‐TKIs, the clinical and imaging findings of this case were consistent with TAPOs. For TAPOs occurring because of MET‐TKI, the drug can be continued under careful observation even if GGOs appear.

## Graphical abstract

Mesenchymal epithelial transition factor receptor (MET) tyrosine kinase inhibitors (MET‐TKIs) have been approved for the treatment of non‐small cell lung cancers with MET exon 14 skipping mutations. Here, we report a case wherein TAPOs appeared during the course of treatment with tepotinib, a MET‐TKI. For TAPOs occurring because of MET‐TKI, the drug can be continued under careful observation even if GGOs appear.

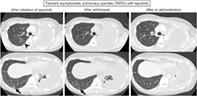

## INTRODUCTION

In recent years, specific gene‐targeting therapies have been developed for the treatment of lung cancer.^1^ Mesenchymal epithelial transition factor receptor (MET) mutations account for 3% of non‐small cell lung cancer.^2^ MET mutation‐positive lung cancer is associated with a poorer prognosis than MET mutation‐negative lung cancer;^3^ thus, treatment with MET tyrosine kinase inhibitors (MET‐TKIs) such as tepotinib is of paramount importance as they improve overall survival in patients with MET mutation‐positive lung cancer.^4^ In the VISION study, tepotinib‐induced interstitial lung disease (ILD) was suspected in six of 152 patients^5^ with a frequency similar to that of osimertinib, an epidermal growth factor receptor (EGFR)‐TKI.^6^ A few studies have reported osimertinib‐associated transient asymptomatic pulmonary opacities (TAPOs) as a benign feature.^7^, ^8^, ^9^ However, tepotinib‐associated TAPOs have never been reported.

Here, we present a patient who developed ground‐glass opacities (GGOs) after the initiation of tepotinib and therefore was suspected to have TAPOs.

## CASE REPORT

**FIGURE 1:**
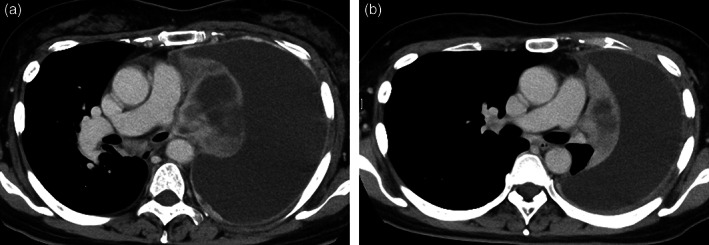
Tepotinib was administered with a reduction in tumor size and decrease in pleural effusion. (a) Chest computed tomography (CT) immediately prior to starting tepotinib. (b) Chest CT performed 43 days after the initiation of tepotinib showing shrinkage in tumor size and decrease in pleural effusion

**FIGURE 2:**
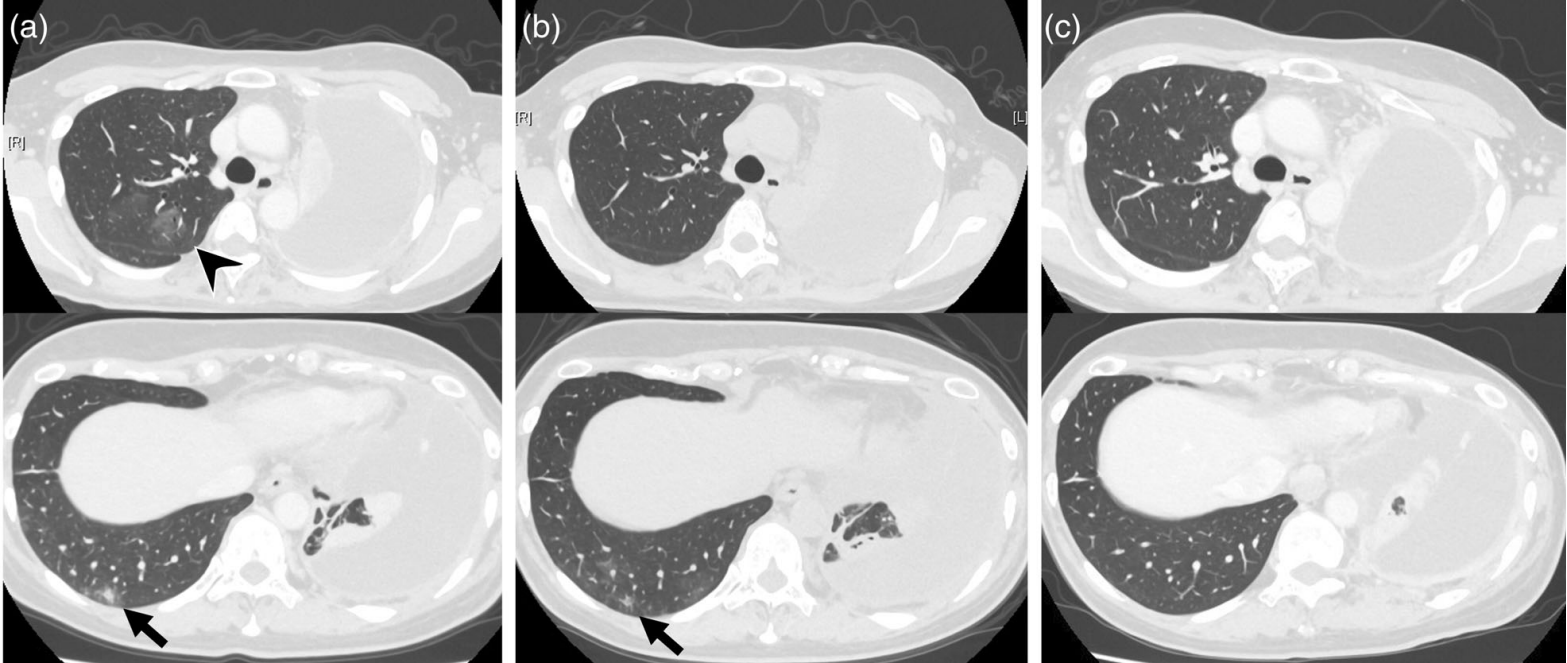
Series of chest computed tomography images. (a) Ground‐glass opacities (GGOs) in the right upper lobe (arrowhead) and a subpleural nodule in the right lower lobe (arrow) after initiation of tepotinib. (b) GGOs disappeared and the subpleural nodule (arrow) appeared to be fading 3 weeks after withdrawal of tepotinib. (c) GGOs and the subpleural nodule disappeared completely 5 months after drug readministration

A 57‐year‐old female patient with no history of smoking and unremarkable medical history was admitted to our hospital with complaints of cough, dyspnea, and chest pain for 1 month. Chest X‐ray revealed left pleural effusion. Chest computed tomography (CT) showed a mass in the left lower lobe, mediastinal lymph node metastasis, and right pleural effusion. Pleural fluid cytology led to the diagnosis of adenocarcinoma, and the clinical diagnosis was lung adenocarcinoma with stage T3N2M1a (Union for International Cancer Control, eighth edition). Three weeks later, the patient complained of worsening dyspnea, and a chest radiograph revealed increased left pleural effusion with a mediastinal shift. Owing to rapid progression, the patient was treated with a combination of cisplatin and pemetrexed as first‐line therapy before the multigene results were available. Subsequently, the patient was switched to oral 500 mg tepotinib (450 mg active moiety) once daily after the multigene test revealed MET exon 14 skipping mutation. A chest CT performed 43 days after the start of tepotinib showed a partial response of lung cancer (Figure 1a, b), but incidentally showed GGOs in the right upper lobe and a subpleural nodule in the right lower lobe (Figure 2a). There were no symptoms of cough or dyspnea. A polymerase chain reaction (PCR) test for severe acute respiratory syndrome coronavirus 2 (SARS‐CoV‐2) on nasopharyngeal swabs was negative. Despite the symptom‐free status of the patient, the administration of tepotinib was discontinued. This was due to severe impairment of lung function in the left lung caused by lung cancer and pleural effusion (Figure 1b), which could potentially result in severe respiratory failure if the GGOs in the right lung were to progress. Three weeks after withdrawal, chest CT revealed the disappearance of shadows (Figure 2b). Therefore, oral tepotinib was resumed at a reduced dose of 250 mg (225 mg active moiety) once daily. No recurrence of GGOs was confirmed on chest CT for 5 months after drug resumption (Figure 2c).

## DISCUSSION

Ground‐glass opacities that manifest during lung cancer treatment are suspected to be indicative of tumor progression or drug‐induced ILD. Although TKI‐induced ILD is rare, it is considered one of the most lethal adverse events, and MET‐TKI‐induced ILDs have also been reported.^5^ Noonan et al. reported a high frequency of TAPOs during osimertinib therapy, and TAPO was found in 15 out of 74 patients (20.3%).^7^ TAPOs are considered a benign feature of osimertinib therapy that can be confused with pulmonary progression or the onset of severe ILD; if GGOs are asymptomatic and localized, assuming no tumor progression, these GGOs are considered as TAPOs and osimertinib treatment is continued under careful observation as a promising option.

TAPOs associated with MET‐TKIs have not been previously reported. However, this case was asymptomatic, GGOs were localized, and the tumor did not show any enlargement; rather, it shrunk in line with TAPOs. In addition, the CT images of this case had imaging patterns similar to TAPOs associated with osimertinib treatment, such as GGOs and subpleural nodules.^7^

As a limitation, tepotinib‐induced ILD was also suspected owing to the appearance of shadows after initiation of tepotinib in this case. In general, pulmonary toxicity, such as ILD, is not associated with dose intensity,^10^ making it difficult to reinitiate the same TKI. There are several reports of readministration of the same EGFR‐TKI in patients with suspected EGFR‐TKI‐induced ILD, all accompanied by steroid use.^11^, ^12^, ^13^ There are reports of patients with MET‐TKI‐induced ILD who are able to switch to a different MET‐TKI and restart treatment.^10^, ^14^ However, the patient in this case was able to continue on the same MET‐TKI, albeit at a reduced dose, without steroid use. Although the possibility of TKI‐induced ILD could not be completely ruled out in this case, we considered TAPOs to be the most likely cause of GGOs. For further understanding of these events, prospective data collection, including other MET‐TKIs, would be helpful.

We encountered a case in which TAPOs were suspected, rather than drug‐induced ILD, during MET‐TKI treatment. Readministration of MET‐TKIs at a reduced dosage under close observation was thought to be the appropriate alternative.

## AUTHOR CONTRIBUTIONS

Writing – Original Draft, S.I.; Writing – Review & Editing, N.Y., Y.S., Y.H., A.S., T.I., T.N.; Supervision, S.I.

## CONFLICT OF INTEREST

The authors declare no conflict of interests.
